# First Molecular Identification of Trypanosomes and Absence of *Babesia* sp. DNA in Faeces of Non-Human Primates in the Ecuadorian Amazon

**DOI:** 10.3390/pathogens11121490

**Published:** 2022-12-07

**Authors:** Gabriel Carrillo-Bilbao, Juan-Carlos Navarro, Sarah Martin-Solano, María-Augusta Chávez-Larrea, Cristina Cholota-Iza, Claude Saegerman

**Affiliations:** 1 Research Unit of Epidemiology and Risk Analysis Applied to Veterinary Sciences (UREAR-Uliège), Fundamental and Applied Research for Animal and Health (FARAH) Center, Department of Infections and Parasitic Diseases, Faculty of Veterinary Medicine, University of Liège, 4000 Liège, Belgium; 2 Facultad de Filosofía y Letras y Ciencias de la Educación, Universidad Central del Ecuador, Quito 170521, Ecuador; 3 Instituto de Investigación en Zoonosis (CIZ), Universidad Central del Ecuador, Quito 170521, Ecuador; 4 Grupo de Investigación en Enfermedades Emergentes, Ecoepidemiología y Biodiversidad, Facultad de Ciencias de la Salud, Universidad Internacional SEK, Quito 170134, Ecuador; 5 Grupo de Investigación en Sanidad Animal y Humana (GISAH), Departamento de Ciencias de la Vida y la Agricultura, Carrera Ingeniería en Biotecnología, Universidad de las Fuerzas Armadas-ESPE, P.O. Box 171-5-231, Sangolquí 171103, Ecuador

**Keywords:** DNA, non-human primate, *Trypanosoma*, *Babesia*, faecal samples, Ecuadorian Amazon, wildlife rehabilitation center

## Abstract

Trypanosomes are a group of pathogens distributed in the continents of Africa, America, Asia and Europe, and they affect all vertebrates including the neotropical primate group. Information about the trypanosome’s diversity, phylogeny, ecology and pathology in non-human primates (NHPs) from the neotropical region is scarce. The objective of the study was to identify *Trypanosoma* and *Babesia* molecularly in NHPs under the phylogenetic species concept. We extracted DNA from a total of 76 faecal samples collected between 2019 and 2021, from a total of 11 non-human primate species of which 46 are from captive NHPs and 30 are free-living NHPs in the Western Amazon region of Ecuador. We did not detect DNA of *Babesia* sp. by polymerase chain reaction test in any of the faecal samples. However, the nested-PCR-based method revealed *Trypanosoma* parasites by ITS gene amplification in two faecal samples; one for the species *Leontocebus lagonotus* (from the captive population) and a second one for *Cebus albifrons* (from the free-ranging population). Maximum parsimony and likelihood methods with the Kimura2+G+I model inferred the evolutionary history of the two records, which showed an evolutionary relationship with the genus *Trypanosoma*. Two sequences are monophyletic with *Trypanosoma.* However, the number of sequences available in GenBank for their species identification is limited. The two samples present different molecular identifications and evolutionary origins in the tree topology. We are most likely referring to two different species, and two different localities of infection. We suggest that health management protocols should be implemented to prevent the transmission of blood-borne pathogens such as *Trypanosoma* sp. among captive populations. In addition, these protocols also protect the personnel of wildlife rehabilitation centers working in close proximity to NHPs and vice versa.

## 1. Introduction

Neotropical non-human primates (NHPs) are threatened by habitat loss or habitat fragmentation (agricultural activities, logging, oil drilling, new road networks), hunting and the wildlife trade [1,2,3,4]. These activities increase contact between people and NHPs (increase the human–wildlife interface), but also enhance the prevalence of pathogens [5,6,7,8]. Captive and free-ranging neotropical NHPs harbour a large diversity of pathogens [9]. Several factors influence the richness, prevalence, and transmission of pathogens in NHPs. Some individuals are more susceptible than others depending on their sex, age, NHP species, behaviour and social status, population density and geographic location.

Trypanosomes are a widespread group of pathogens that infect all kinds of vertebrates across America, Africa, Asia and Europe [10,11,12,13,14,15]. Trypanosomes are intracellular or extracellular pathogens found in blood, lymph and tissues [16,17,18,19]. Although trypanosomatids are widespread pathogens of mammals in the Americas, their biology, taxonomy and pathology are not well known for neotropical NHPs [20]. Natural infections of trypanosomes are common in neotropical NHPs. The prevalence of trypanosomes among NHPs can vary from one species to another [21]. According to Aysanoa et al. [22], prevalence was higher in free-ranging NHPs than in captive NHPs [23]. Some species such as *Trypanosoma cruzi* are common among NHPs’ trypanosomes and can cause myocarditis, haemorrhage, and encephalitis to NHPs [24,25,26]. In addition to *Trypanosoma cruzi*, NHPs can be infected by 12 more species (Appendix A). Some species are non-pathogenic to the host such as *Trypanosoma minasense* [27]. *T. minasense* does not infect blood-sucking triatomines such as other species of trypanosomes [28], and information of vector is scarce [29]. However, it is suggested that some species of *Trypanosoma* sp. can be transmitted by oral contamination [30,31]. *Trypanosoma minasense*, *T. devei* and *T. lambrechti* are considered the most primitive species infecting neotropical mammals among the other species belonging to the genus *Trypanosoma* [32].

*Babesia* is the genus of several species of tick-borne pathogens affecting the red cells of several mammals and birds [33,34,35,36,37,38,39,40]. Three species of *Babesia* sp. are zoonotic: *B. divergens*, *B. duncani* and *B. microti*. *Babesia microti* in America is a blood pathogen, this apicomplexan can infect humans (serve as accidental host) and causes babesiosis. Rodents are natural reservoirs [41], and ticks of the family Ixodidae that infect deer are the primary vectors of *Babesia microti* [42,43,44]. In old-world NHPs, *Babesia microti* has been observed in several species of NHPs [45,46]. In neotropical NHPs, *Babesia* sp. can be found at least in five different NHP species [47,48]. However, the species of *Babesia* sp. are uncharacterised [49].

The internal transcribed spacers (ITS) are widely used for molecular characterization and phylogenetic studies [50,51,52,53]. The ITS region includes the ITS1 and the ITS2. These ITS vary in size between species and subspecies [54] and show more variation than the ribosomal coding region, with high evolutionary rates [55]. This gene was used to molecularly characterize trypanosomes and to study the phylogenetic of trypanosomes [56,57,58,59,60,61,62]. The Cathepsin L-like (CatL-like) protein is a cysteine protease important in the life cycle and pathogenicity of trypanosomes, involved in mechanisms such as tissue damage, invasion, and recovery of metabolites from host proteins [63,64,65,66]. The active site sequence of the gene encoding this protein, being a conserved, multi-copy gene, has been widely used as a targetable marker for *Trypanosoma* spp. diagnosis, in some cases in combination with other molecular markers such as ITS and 18S [67,68,69,70,71].

Molecular genetic analysis has had its limitations in wild populations due to the difficult access to blood or tissue samples for DNA extraction [72], which is why non-invasive sampling has been chosen worldwide. The main drawback is mainly due to the low amount of genetic material obtained [72]. However, in 1990, Boom et al. [73] presented the first study that succeeded in isolating DNA from epithelial cells that were mixed with faeces. Since then, with improvements included in their protocols, conservation genetics studies use faecal samples for DNA extraction [74,75,76]. Molecular analysis of faecal samples has already been used to decipher the origins of major pathogens in human and non-human primates [77,78,79,80,81,82,83,84,85,86,87], even in blood pathogens [61,88,89]. Successful amplification of *Trypanosoma* sp. [90] and *Babesia* sp. DNA in previous studies from stool samples in mice, dogs, and foxes [91] offers a non-invasive option as a valid alternative to traditional sampling methods.

In Ecuador, information regarding blood pathogens in NHPs is scarce. Therefore, this study aimed for the first time to detect *Trypanosoma* and *Babesia* in captive and free-ranging NHPs from the Ecuadorian Amazon through molecular techniques (PCR and sequencing). 

## 2. Materials and Methods

### 2.1. Sampling Location

**Figure 1:**
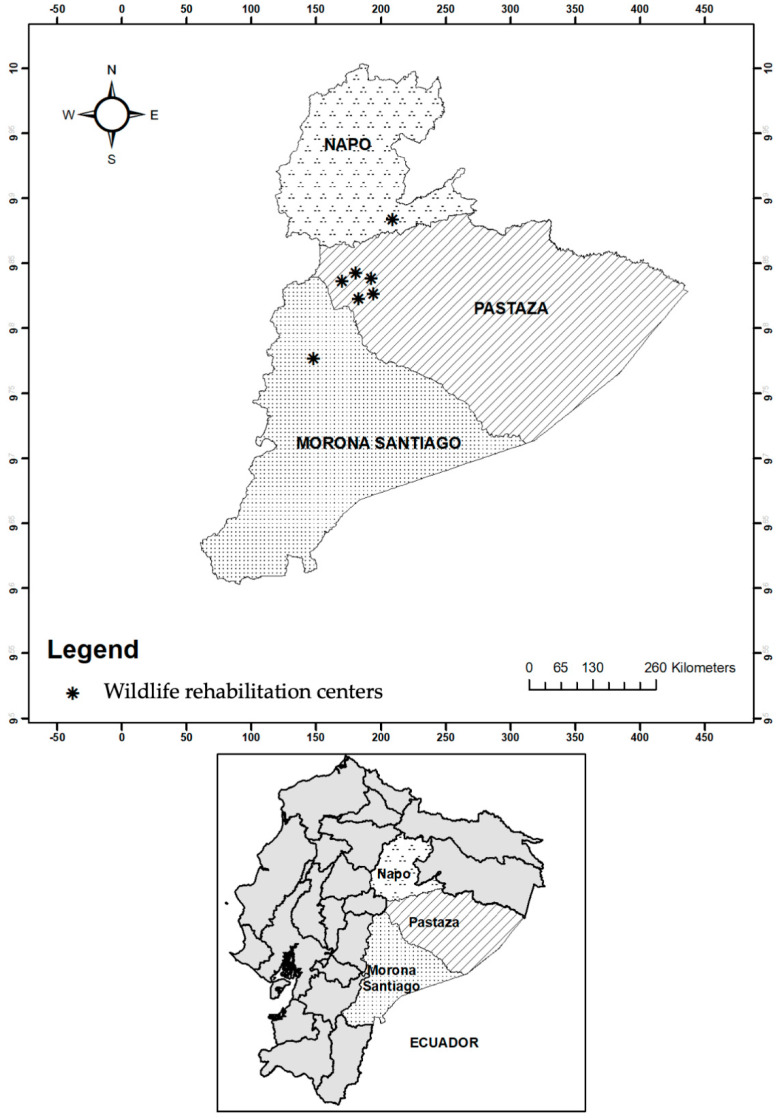
Sampling location of wildlife rehabilitation centers with non-human primates in Ecuador.

This study was performed in Puyo (1°2′9′1.3′ S 78°0.154′ W) and Mera (0°10′0″ S and 78°28′0″ W), Tena (Napo) (0.9938° S 77.8129° W), and Macas (Morona Santiago) (2.3087° S 78.1114° W), four cities in the Western Amazon region. We collected samples from a free-ranging population in Misahualli, Tena (1°2′7.0′ S, 77°39′59.4″ W) and from captive individuals located in 5 different wildlife rehabilitation centers of Ecuador. Captive NHPs have been donated by families or confiscated by the police during roadside checks; individual information such as location origin is uncertain (Figure 1).

### 2.2. Sample Collection and Ethics Statement

We collected a total of 76 fecal samples of 11 species of NHPs (Table 1) between 2019 and 2021. The sample collection and ethical procedures were approved by the local authorities, the Ministerio del Ambiente, Agua y Transición Ecológica, MAATE (No. MAE-DNB-CM-2015-0028-M-002). Individuals were followed daily from 08:00 h to 18:00 h to avoid multiple sampling. In addition, all animals were individually identified to avoid confusion between individuals and to facilitate species, sex and age association [92]. Finally, we collected the faecal samples immediately after defecation to avoid a possible contamination from the environment and were taken at least 24 h without disturbing the animals. In primatology, when birth dates are unknown, age as well as sex, is generally assigned in categories based on physical characteristics including body size, dentition and gland development in species where these are evident, and behavioral characteristics [93,94,95]. All samples were processed at the International Centre of Zoonoses at the Central University of Ecuador and examined at the Biotechnology Animal Lab at the Universidad de las Fuerzas Armadas ESPE.

### 2.3. Storage and DNA Isolation Protocol

For the molecular evaluation, samples were stored in 50 mL Falcon tubes in 99% alcohol at −20 °C to prevent the degradation of DNA. In addition, 600 μL of faeces suspension (1:3; 1 part of faecal sample and 3 parts of ethanol 96–100%) was centrifuged for 2 min at 239 g and the pellet was washed with 1 mL of PBS Buffer (Oxoid, Hampshire, England). This solution (pellet + PBS) was centrifuged for 5 min and the supernatant was discarded. This washing step was repeated three times. Next, the pellet was resuspended in 600 μL of 2% PVPP (polyvinylpolypyrolidone—Sigma), and frozen overnight at −20 °C to facilitate the capture of phenols in the sample. DNA extraction was performed twice on different days using the QIAamp Stool FAST Mini Kit (Qiagen GmbH, Hilden, Germany) following the manufacturer’s instructions. To prevent cross-contamination, sample preparation, DNA extraction, and the polymerase chain reaction (PCR) were performed in completely different and separate rooms.

### 2.4. Molecular Amplification and Sequencing of Trypanosoma *sp.*

The molecular identification was performed with two PCR assays: (i) A CatL-PCR, according to reaction conditions by Cortez et al. [70] (Table 2), with adaptations in the reaction mixture, that consisted of a final volume of 25 μL with 1× of Buffer, 1 μM of each primer DTO154 and DTO155 (Table 3), 1.5 mM of ${\mathrm{MgCl}}_{2}$, 0.2 mM of dDNT Mix, 1.25 U of Taq Platinum Polymerase (Invitrogen) and 2 μL of DNA. (ii) A nested ITS-PCR as described by [96,97], the reaction mixture consisted of 1× of Buffer, 1 μM of each primer (ITS1 and ITS2 in the first reaction, ITS3 and ITS4 in the second reaction) (Table 3), 2.5 mM of ${\mathrm{MgCl}}_{2}$, 0.2 mM of dDNT Mix, 1.25 U of Tap Platinum Polymerase (Invitrogen) and 2 μL of DNA in the first reaction and 1uL of first reaction PCR product in the second reaction; the amplification consisted of an initial denaturation of 95 °C for 7 min; 35 cycles of 95 °C for 1 min, 59 °C for 1 min, 72 °C for 2 min in the first reaction and 1.5 min in the second reaction; and a final extension step at 72 °C for 10 min (Table 4). The final PCR products of the CatL-PCR and ITS-PCR were observed using the electrophoresis of an agarose gel under UV light. ITS-PCR amplicons were cut, extracted using the Wizard^®^ SV Gel and PCR Clean-Up System (Promega) and sequenced (Sanger sequencing) by Macrogen (South Korea). Every PCR reaction contained a negative (nuclease-free water) and a positive control. A positive control for CatL-PCR was a positive DNA sample of *Trypanosoma vivax* [98] and for ITS-PCR a positive DNA sample of *Trypanosoma theileri*, available in the Laboratorio de Biotecnología Animal of the Universidad de las Fuerzas Armadas ESPE. Two sequences belonging to the 5.8S and ITS-2 were recovered from Sanger sequencing Macrogen Korea.

### 2.5. Molecular Amplification and Sequencing of Babesia *sp.*

The molecular identification by PCR was performed with the primers designed by Olmeda et al. [99] and using the reaction condition described by Medina Naranjo et al. [100], which consisted of: 1× of Buffer, 0.25 μM of each primer Piro A and Piro B (Table 3), 1.5 mM of ${\mathrm{MgCl}}_{2}$, 0.2 mM of dDNT Mix, 0.5 U of Tap Platinum Polymerase (Invitrogen) and 2 μL of DNA; the amplification of an initial denaturation of 94 °C for 5 min; 35 cycles of 94 °C for 1 min, 55 °C for 1 min, 72 °C for 30 sec; and a final extension step at 72 °C for 5 min (Table 5). The PCR products were observed using the electrophoresis of an agarose gel under UV light. Every PCR reaction contained a negative and a positive control. A positive control for *Babesia* sp. was a DNA sample obtained from the study performed by Chávez-Larrea et al. [101].

### 2.6. Molecular Analysis: Sequence Assembled, Alignments and Phylogenetic Analyses

Sequences were uploaded to GenBank under the accession number OP683488.1 for *Trypanosoma* sp. detected in *Leontocebus lagonotus* and OP683532.1 for *Trypanosoma* sp. detected in *Cebus albifrons*. Our two 5.8S-ITS-2 sequences of 400 bp length were contig assembly and consensus sequences of ITS-2 were performed and edited using Assembler by MacVector software 18.2.5 [9], then the sequence identity was confirmed by BLAST in NCBI resources. The two sequences were first aligned with the unique complete sequence 18S-ITS1-5.8S-ITS2-28S available in GenBank that included ITS2 (*T. minansense* AB362411.1 by Sato et al. [102], recovered from new-world NHPs from South American tamarins) to corroborate the portion of rDNA and matching or sequences (from 2636 bp to 3019 bp). The 5.8S-ITS-2 sequences deposited in GenBank NCBI from other species of *Trypanosoma* were included as sister groups and *Leishmania* was selected as the outgroup *sensu* [103] to get a wide geographic diversity and taxonomic representative and to test the phylogenetic species monophyly.

A phylogenetic analysis was performed using a total of 23 ITS-2 sequences from 13 species of *Trypanosoma* that were retrieved from GenBank and included two species of *Leishmania* as outgroup to corroborate the Blast identity close to *Trypanosomatidae* and to search the evolutionary relationships with other trypanosomes (Table 6).

The DNA sequences were aligned using MacVector 18.2.5 by the ClustalW algorithm with high gap creation and extension penalties by 30.0 and 10.0, respectively, searching for a strong positional homology.

The evolutionary history was inferred by using the maximum parsimony and likelihood methods with the Kimura2+G+I model. Maximum Parsimony analyses were implemented in PAUP 4.0a (169 build) [104] using the heuristic search option with a Tree Bisection Reconnection branch-swapping algorithm with at-random stepwise addition of 10 replicates for each search and 100–1000 replications per analysis. Gaps were treated both as missing data. The characters were treated as unordered, and equally weighted, after the characters were weighted by consistency index. The robustness of the trees was estimated using parsimony bootstrap with 1000 pseudoreplicates after excluding uninformative characters [105]. We also performed a Maximum Likelihood (ML, and substitution model estimated on MEGAX).

## 3. Results

We have performed a PCR and a Nested PCR to detect the gene *Trypanosoma* from faecal samples of 11 species of NHPs. The PCR with the Catepsine L-Like gene failed to amplify DNA from *Trypanosoma* sp., although this gene can be easily amplified in other samples from wild mammals [106]. On the other hand, the Nested PCR of 35 cycles successfully amplified the ITS1 gene.

The nested PCR results showed a total of 8/76 (10.53%) samples positive for *Trypanosoma* sp. Positive samples belong to *Alouatta seniculus* (n = 1), *Ateles belzebuth* (n = 1), *Cebus albifrons* (two captive and two free-ranging individuals), *Lagothrix lagotricha* (n = 1) and *Leontocebus lagonotus* (n = 1). Among these positive samples, two (2.63%) yielded amplicons for trypanosomes species in *Leontocebus lagonotus* (from the captive population) and *Cebus albifrons* (from the free-ranging population). The sequences presented a first Blast identity with *Trypanosomatidae*.

We did not observe positive samples for *Babesia* sp. for any of the samples.

From the 458 *Trypanosoma* sequences in GenBank that partially or completely included ITS-2 belonging to 16 species, we aligned the two ITS sequences amplified from the faecal samples with 21 ITS sequences from 13 species (Table 3) (three species from Russia were not included) to elaborate the cladogram.

**Figure 2:**
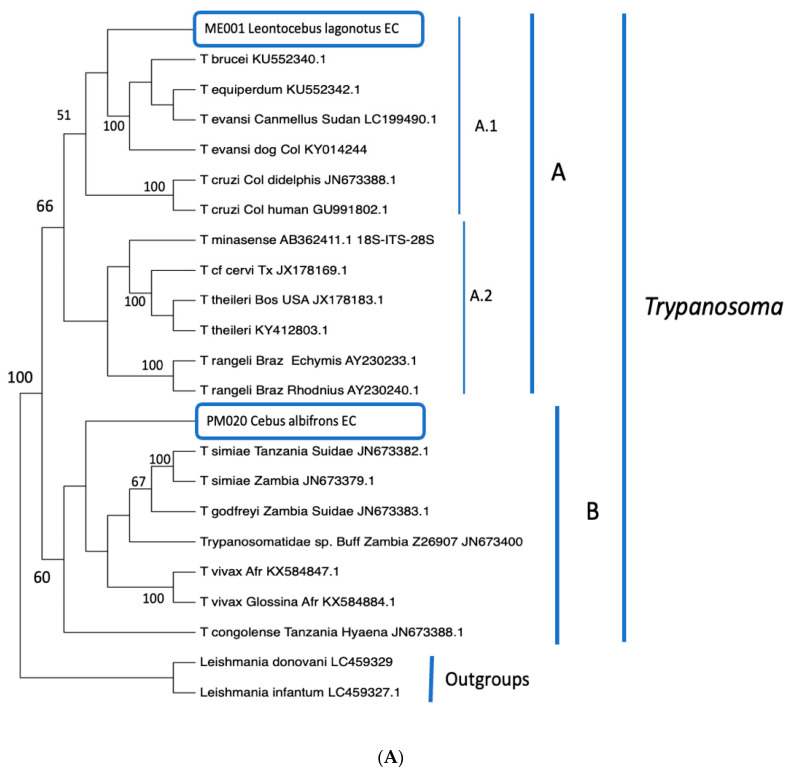

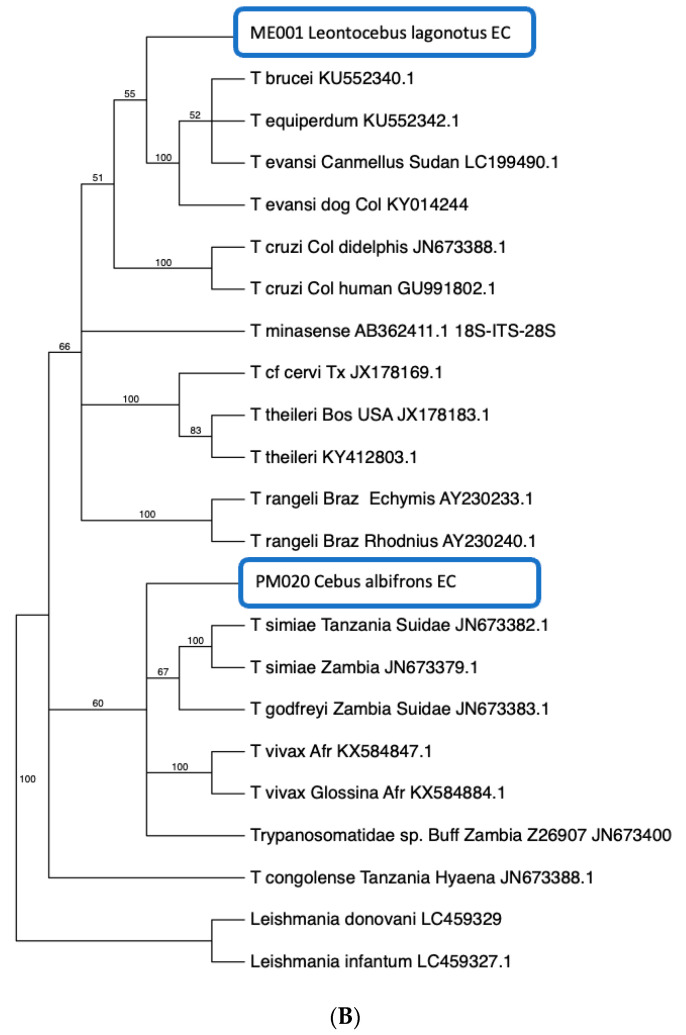
(**A**). Cladogram of the two species of trypanosomes found in NHPs and (**B**). Bootstrap Consensus Tree. Legend: Numbers are percentages of homology. The circled samples are from the study. For (**A**), A and B are clades; B.1 and B.2 are subclades.

The cladogram showed that our two sequences belong to the trypanosomes genus. Our results revealed two unexpected novel sequences. The topology of the cladogram (Figure 2A) shows two clades (A and B). The clade shows subclades A.1 with *T. cruzi* as a sister group of the *Trypanosoma* group of *Leontocebus lagonotus* ME001 Ecuador + *T. brucei*, *T. evansi* and *T. equiperdum* in derived position and subclade A.2 with *T. rangeli* + [*minasense* (cf.*cervi* + *theileri*)]. However, there is no sequence available with which to identify monophyly.

Clade B shows the sequence of *Cebus albifrons* PM020 from Ecuador, internally and closely related to *T. congolense* and basal to *T. vivax* + [(Trypanosmatidae sp (*T. godfreyi* + *T. simiae*)], showing its close relationship with these species. Likewise, there is no ITS-2 sequence in GenBank that shows monophyly with our sequence for a specific identification.

The topologies using maximum parsimony (MP) and maximum likelihood (ML) under the Kimura2+G+I model showed identical relationships (Figure 2B), the two *Leishmania* species as outgroup allow corroborating the monophyly of *Trypanosoma* (100% bootstrap), as well as our sequences showing their evolutionary relationship within the genus.

## 4. Discussion

The present study is the first at the national level and one of the few at the regional level to identify two species of *Trypanosoma* sp. using non-invasive techniques. Although the ITS region was successfully amplified in wild gorillas (*Gorilla gorilla gorilla*) and chimpanzees (*Pan troglodytes troglodytes*) for trypanosomes, this is the first study in the neotropical region to use the ITS region to amplify trypanosomes in faecal samples. We identified the first record of *Trypanosoma* for the NHP species *Leontocebus lagonotus* and the first report for Ecuador of *Trypanosoma* in *Cebus albifrons*. We detected 10.53% of positive samples, whereas only 2.63% yielded a positive sequence. This prevalence is lower than other studies [107,108]. In Aysanoa et al. [22], they found a lower prevalence in captive NHP individuals than in wild individuals. Captive animals may be subject to liberation projects, and they can introduce new trypanosomatids to liberation sites. Triatomines were found in a Brazilian zoo, infesting neotropical NHPs. This indicates that the same pattern could be possible in Ecuadorian wildlife rehabilitation centers where vegetation could facilitate the presence of trypanosomes vectors. Common vectors of trypanosomes like triatomine bugs (*Panstrongylus geniculatus, Triatoma dimidiata, Rhodnius pictipes and Rhodnius robustus*) can be found in the Ecuadorian Amazon [17,109] and the proximity of the forests to the centers would facilitate the maintenance of the forest cycle.

Previous studies have suggested that trypanosomes tend to have harmful effects on the health of infected hosts [110,111,112,113,114]. However, information on the effect of triatomines in NHPs is scarce. The individuals who tested positive had no obvious symptoms that would allow us to make a statement about their health condition, noting that trypanosome records have been made in healthy individuals as well as in sick individuals.

After phylogenetic reconstruction, we identified two large groups: the first, in which we found the sample of *Leontocebus lagonotus* within the same cladogram as the species of *T. brucei*, *T. equiperdum*, *T. evansi*, *T. cruzi*, *T. minasense* and *T. theileri*. In the second cladogram, we found the sample of *Cebus albifrons* together with the species of: *T. simiae*, *T. godfreyi*, *T. vivax* and *T. congolense*. This distribution coincides with several authors [102]. The two samples show different molecular identifications and evolutionary origins, certainly two species, and two localities of infection.

The use of molecular tools for the detection of *Trypanosoma* spp. is crucial because of the unreliability of detection methods based on the observation of their morphology [115,116]. The two sequences are shown to be in monophyly with *Trypanosoma;* however, there are not enough sequences available in GenBank for their specific identification.

As mentioned before, the gene from the Cathepsin L-like protein failed to amplify in the faecal samples. However, past studies diagnosed *T. rangeli*, *T. cruzi* and *T. theileri* with this gene [71,106,117,118]. This protein is a lysosomal cysteine proteinase. The Cathepsin L-like protein is found in several stages of cell multiplication and differentiation as well as cell metabolism and virulence (host cell invasion, immune evasion) in protozoan parasites such as trypanosomes [119,120]. However, according to Cortez et al. [70], this gene has a different number of copies depending on the trypanosome species and therefore is species-specific, and because we were surveying trypanosomes in general, the protocol failed to amplify for all trypanosome species and specifically for neotropical NHPs’ trypanosomes, given what was observed with the amplification of the ITS gene.

Based on the findings, the ITS gene is a useful molecular marker to detect trypanosomes; however, for further studies, it is suggested to amplify with 18S-ITS-1 (higher availability); this combination would support us in defining in more specific detail the molecular characterization of these two records. Unfortunately, only *T. simiae*, *T. rangeli*, *T. cruzi* and *T. minasense* (included) of the NHPs-associated *Trypanosoma* have ITS2 sequences available in GenBank from the list in Appendix A.

We did not record the presence of *Babesia* sp., a protozoan pathogen with a worldwide distribution restricted to tropical and subtropical areas [101]. Non-human primates are a group of mammals that have generated strategies to prevent pathogens [121,122,123]. One of these strategies is grooming. Grooming is a behaviour that directly supports health-related aspects of different primate species, including the removal of ectopathogens such as leeches in *Macaca fuscata* [124] and ticks [125,126]. For *Papio cynocephalus* (Africa), it was recorded that the amount of grooming received, sex, age and hierarchical level affected the tick load of an individual. However, the primary function of grooming contributes to social aspects in different old=world primate species [127], whereas, in new-world primates, it is suggested that the main function of grooming is hygienic [128]. For this reason, this type of grooming can explain the absence of *Babesia* sp. in our study. It is important to conduct long-term studies that allow us to relate the presence of ticks to the prevalence/absence of tick-borne diseases in non-human primates.

## 5. Conclusions

This is the first study to amplify trypanosomes in Ecuadorian NHP species.

Even if the prevalence was low, we suggest the implementation of health management protocols to avoid the transmission of blood-borne pathogens such as *Trypanosoma* sp. among captive populations. In addition, these protocols protect the personnel of wildlife rehabilitation centers working in close proximity to NHPs and vice versa. Socioecological aspects are of utmost importance to understand pathogen–vector–host relationships in different species of NHPs. In Ecuador, research activities should be focused on blood pathogens to fill the gap of information and to implement surveillance programs with regular and effective monitoring protocols adapted to NHPs. We suggest to increase the monitoring of free-ranging groups across Ecuador to clarify the role of NHPs as reservoir hosts of novel trypanosomes.

## Figures and Tables

**Table 1:** Non-human primate’s species screened for *Trypanosoma* sp. and *Babesia* sp.

Habitat	Non-Human Primate Species	n	Sex		Age		
			Male	Female	Juvenile	Subadult	Adult
Free-ranging	*Cebus albifrons*	18	8	10	7	4	7
Captive	*Alouatta seniculus*	4	0	4	2	1	1
	*Ateles belzebuth*	4	4	0	2	1	1
	*Aotus vociferans*	1	0	1	0	0	1
	*Cebuella pygmaea*	2	1	1	1	0	1
	*Cebus apella*	1	0	1	0	0	1
	*Cebus albifrons*	10	9	1	3	1	6
	*Lagothrix lagotricha*	18	7	11	0	6	11
	*Leontocebus lagonotus*	9	4	5	0	0	9
	*Plecturocebus discolor*	4	3	1	3	0	1
	*Saimiri sciureus*	3	3	0	1	1	1
	*Sapajus apella*	2	1	1	1	0	1

**Table 2:** Three steps CatL-PCR cycles, temperature, and time for *Trypanosoma* sp.

Step		Temperature	Time	Number of Cycles
Step 1	Pre-denaturation	94 °C	5 min	1 cycle
Step 2	Denaturation	94 °C	1 min	35 cycles
	Annealing	56 °C	1 min	
	Extension	72 °C	1 min	
Step 3	Final extension	72 °C	5 min	1 cycle

**Table 3:** Sequences of the primers for *Trypanosoma* sp. and *Babesia* sp.

	Reaction	Primer	Oligonucleotide Sequence	
Trypanosoma	CatL-PCR	DTO154	5′-ACAGAATTCCAGGGCCAATGCGGCTCGTGCTGG-3′	Forward
		DTO155	5′-TTAAAGCTTCCACGAGTTCTTGATGATCCAGTA-3′	Reverse
	ITS-PCR First Reaction	ITS1	5′-GATTACGTCCCTGCCATTTG-3′	Forward
		ITS2	5′-TTGTTCGCTATCGGTCTTCC-3′	Reverse
	ITS-PCR Second Reaction	ITS3	5′-GGAAGCAAAAGTCGTAACAAGG-3′	Forward Reverse
		ITS4	5′-TGTTTTCTTTTCCTCCGCTG-3′	
Babesia		Piro A	5′-AATACCCAATCCTGACACACAGGG-3′	Forward Reverse
		Piro B	5′-TTAAATACACGAATGCCCCCCCAAC-3′	

**Table 4:** Three steps nested ITS-PCR cycles, temperature, and time for *Trypanosoma* sp.

Step		Temperature	Time	Number of Cycles
Step 1	Pre-denaturation	95 °C	7 min	1 cycle
Step 2	Denaturation	95 °C	1 min	35 cycles
	Annealing	59 °C	1 min	
	Extension	72 °C	2 min (first reaction) 1.5 min (second reaction)	
Step 3	Final extension	72 °C	10 min	1 cycle

**Table 5:** Three steps PCR cycles, temperature, and time for *Babesia* sp.

Step		Temperature	Time	Number of Cycles
Step 1	Pre-denaturation	94 °C	5 min	1 cycle
Step 2	Denaturation	94 °C	1 min	35 cycles
	Annealing	55 °C	1 min	
	Extension	72 °C	30 s	
Step 3	Final extension	72 °C	5 min	1 cycle

**Table 6:** Sequences obtained from GenBank to elaborate the cladograms.

Species/Sequences	GenBank ID
*Leishmania infantum* (outgroup)	LC459327.1
*Leishmania donovani* (outgroup)	LC459329
*Trypanosoma vivax*	KX584847.1; KX584884.1
*T evansi*	LC199490.1; KY014244
*T theileri*	KY412803.1; JX178183.1
*T congolense*	JN673388.1
*T simiae*	JN673382.1; JN673379.1
*T cruzi*	JN673388.1; GU991802.1
*T minasense*	AB362411.1
*T brucei*	KU552340.1
*T godfreyi*	JN673383.1
*T equiperdum*	KU552342.1
*T rangeli*	AY230233.1; AY230240.1
*Trypanosomatidae sp.*	JN673400
*T* cf. *cervi*	JX178169.1

## Data Availability

The data that support the findings of this study are available from the corresponding author upon request.

## Appendix A

Trypanosomes in neotropical non-human primate species (updated from Carrillo-Bilbao et al. [129]).

**Table:**

Trypanosome	Non-Human Primate Species	Reference
*Leishmania* sp. *Leishmania (Viannia)*	*Alouatta guariba* Atelidae (unknown species) *Aoutus azarai azarai* *Aotus nigriceps* *Callicebus nigrifrons* *Callithrix jacchus* *Callithrix penicillata* *Cebus macrocephalus* *Lagothrix cana* *Leontopithecus crysomelas* *Pithecia* sp. *Pithecia irrorata* *Saguinus imperator* *Saimiri ustus madeirae* *Sapajus apella* *Sapajus xanthosternos*	[130,131,132,133,134]
*Leishmania amazonensis*	*Ateles paniscus*	[135]
*Leishmania braziliensis*	*Saguinus geoffroyi*	[136]
*Leishmania chagasi* *Leishmania infantum*	*Callicebus nigrifrons* *Callithrix jacchus*	[133] [137]
*Leishmania mexicana*	*Alouatta palliata* *Alouatta pigra*	[138]
*Leishmania (Viannia) shawi*	*Chiropotes satanus* *Sapajus apella*	[139]
*Trypanosoma* sp.	*Alouatta palliata* *Alouatta seniculus* *Ateles paniscus* *Pithecia pithecia* *Saguinus leucopus* *Saimiri sciureus*	[27,140]
*Trypanosoma cruzi*	*Alouatta palliata* *Alouatta pigra* *Alouatta caraya* *Alouatta seniculus* *Ateles belzebuth* *Ateles chamek* *Ateles geoffroyi* *Ateles fusciceps* *Ateles paniscus* *Aotus sp.* *Aotus azarai* *Aotus nigriceps* *Cacajao calvus* *Callicebus personatus* *Callicebus nigrifrons* *Callithrix geoffroyi* *Callithrix jacchus* *Callithrix penicillata* *Cebuella pygmaea* *Cebus albifrons* *Cebus capucinus* *Cebus olivaceus* *Cheracebus torquatus* *Chiropotes satanas* *Lagothrix lagotricha* *Lagothrix cana* *Leontopithecus chrysopygus* *Leontopithecus chrysomelas* *Leontopithecus rosalia* *Leontocebus fuscicollis* *Leontocebus fuscicollis weddelli* *Leontocebus nigricollis* *Mico chrysoleucus* *Mico argentatus* *Mico emiliae* *Pithecia irrorata* *Pithecia chrysocephala* *Plecturocebus brunneus* *Saguinus niger* *Saguinus geoffroyi* *Saguinus bicolorbicolor* *Saguinus imperator imperator* *Saguinus labiatus* *Saguinus leucopus* *Saguinus midas* *Saguinus mystax* *Saguinus ustus* *Saimiri boliviensis* *Saimiri sciureus* *Saimiri ustus* *Sapajus apella* *Sapajus flavus* *Sapajus libidinosus* *Sapajus robustus* *Sapajus xanthosternos*	[21,26,31,32,58,107,108,136,138,141,142,143,144,145,146,147,148]
*Trypanosoma devei*	*Cebuella pygmaea* *Callimico goeldii* *Leontocebus fuscicollis weddelli* *Leontocebus tamarin* *Saguinus imperator*	[108,149,150]
*Trypanosoma diasi*	*Alouatta palliata* *Sapajus apella apella*	[27,149]
*Trypanosoma forestali*	*Alouatta guariba* *Alouatta caraya*	[32]
*Trypanosoma hippicum*	*Alouatta guariba* *Alouatta seniculus*	[151]
*Trypanosoma lambrechti*	*Alouatta seniculus* *Cebus albifrons* *Cheracebus torquatus* *Chiropotes satanas* *Pithecia pithecia* *Sapajus apella*	[32,136,151]
*Trypanosoma lesourdi*	*Ateles paniscus*	[32]
*Trypanosoma mycetae*	*Alouatta belzebul* *Alouatta belzebul belzebul* *Alouatta palliata* *Alouatta caraya* *Alouatta seniculus* *Chiropotes satanas*	[32,144,148,149,150,151]
*Trypanosoma minasense*	*Alouatta belzebul* *Alouatta caraya* *Alouatta guariba* *Alouatta palliata* *Alouatta seniculus* *Aotus trivirgatus* *Ateles fusciceps* *Ateles geoffroyi griscescens Callithrix jacchus* *Callithrix penicillata* *Cebus albifrons* *Cebus capucinus* *Leontocebus weddelli* *Leontocebus fuscicollis weddelli* *Plecturocebus ornatus* *Saguinus geoffroyi* *Saguinus imperator imperator* *Saguinus midas* *Saimiri sciureus* *Saimiri sciureus macrodon* *Saimiri ustus* *Sapajus apella*	[21,27,32,102,108,136,141,144,148,149,152,153]
*Trypanosoma rangeli like*	*Alouatta seniculus* *Alouatta belzebul* *Alouatta caraya* *Cebuella pygmaea* *Cebus albifrons unicolor* *Cebus capucinus* *Callimico goeldii* *Leontocebus fuscicollis weddelli* *Pithecia pithecia* *Saguinus bicolor* *Saguinus bicolor bicolor* *Saimiri boliviensis* *Saimiri ustus* *Saimiri sciureus* *Saguinus geoffroyi* *Saguinus imperator imperator* *Saguinus midas* *Saimiri boliviensis* *Sapajus apella Sapajus libidinosus*	[21,30,108,141,144,148,154,155]
*Trypanosoma saimiri*	*Saimiri sciureus sciureus*	[150]
*Trypanosoma venezuelensis*	*Alouatta guariba* *Alouatta seniculus*	[151]
